# Optofluidic Force Induction for Online Monitoring of Particle Size Distributions in Emulsion Polymerization Reactions

**DOI:** 10.1021/acspolymersau.5c00061

**Published:** 2025-10-02

**Authors:** Usue Olatz Aspiazu, Marko Šimić, Michael Schnur, Ulrich Hohenester, Christian Hill, Doris Auer, Maria Paulis, Jose Ramon Leiza

**Affiliations:** † POLYMAT, Kimika Aplikatua Saila, Kimika Fakultatea, University of the Basque Country (EHU), Joxe Mari Korta Zentroa, Donostia-San Sebastián 20018, Spain; ‡ 592767 BRAVE Analytics GmbH, Stiftingtalstraβe 14, Graz, Styria 8010, Austria; § Gottfried Schatz Research Center, Division of Medical Physics and Biophysics, Medical University of Graz, Neue Stiftingtalstraβe 2, Graz, Styria 8010, Austria; ∥ Institute of Physics, 27267 University of Graz, Universitätsplatz 5, Graz, Styria 8010, Austria

**Keywords:** OF2i, online monitoring, particle size distribution, emulsion polymerization, off-line techniques

## Abstract

Real-time monitoring of particle size distribution (PSD) during emulsion polymerization is vital for understanding reaction mechanisms and improving process control. Conventional offline methods such as Dynamic Light Scattering (DLS), Transmission Electron Microscopy (TEM), and Capillary Hydrodynamic Fractionation chromatography (CHDF), while accurate, are limited in their ability to capture dynamic changes during the reaction. OptoFluidic Force Induction (OF2i) is a novel optical tweezers-based technology that enables online, *in situ* particle characterization. Although previously applied in pharmaceutical contexts, its utility in polymer reaction monitoring remains untested. This study evaluates the performance of OF2i in stirred-tank reactors during *ab initio* and semibatch emulsion polymerizations of styrene and acrylic comonomers. OF2i successfully tracked the evolution of particle size and PSD throughout various reaction stages, including primary nucleation in micelle-free systems, particle growth in seeded semibatch setups, and secondary nucleation simulated through sequential seed additions. It delivered accurate PSD measurements for particles larger than ∼180 nm in polystyrene latexes and ∼200 nm in (meth)­acrylated copolymer systems, with a current temporal resolution of 10 min governed by the system’s automated dilution interface. PSD results were consistent with those obtained by established offline techniques such as DLS, TEM, and CHDF. The system’s ability to operate without composition-specific calibration and to provide continuous, high-resolution data makes it uniquely suited for monitoring transient dynamics and population shifts in real time—capabilities not achievable with conventional methods.

## Graphical abstract

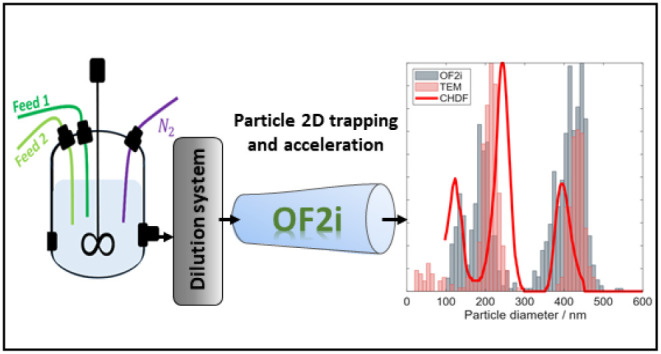

## 1 Introduction

Waterborne polymer dispersions (also known as latexes) are key products in the paint, adhesive, and coating industries, among others, due to their ability to form coherent films. Furthermore, the possibility to obtain *à la carte* characteristics makes these product-by-process materials a very interesting topic with a very wide sort of applications.^1^ Particle size and particle size distribution (PSD) of these latexes are critical characteristics that affect, among others, the film formation behavior and the final properties of the products (e.g., gloss and water sensitivity), as the solid particles that are first dispersed in the continuous medium will have to come together, deform, and diffuse to form the film. Thus, these properties are closely (but not only) related to the size of the particles.^2^ Moreover, the PSD is also crucial during the synthesis process, as it directly affects the viscosity of the latex and thus the heat transfer capacity inside the reactor, which can be exploited for safer reaction procedures.^3–5^

Therefore, monitoring and controlling the PSD (in addition to polymer composition and molar mass distribution) is essential for efficient, sustainable, and reproducible production in latex plants. During the last four decades, academic and industrial researchers have devoted large efforts to developing technologies to monitor in real-time the particle size and PSD. However, an accurate, robust, and simple technology that allows the measurement of the PSD without the need to disturb the emulsion polymerization process is elusive. In a recent review about technologies to measure PSD,^6^ the authors discuss *off-line* (manual sampling and dilution are necessary and at least 60 min of analysis), *online* (sampling, dilution, and analysis are automated, and information about PSD can be retrieved in less than 10 min), and *in-line* (no sampling and dilution is required, analysis is automated, and information is obtained in real-time) technologies. *Online* techniques include off-line techniques adapted for automated sampling and dilution. Within the online techniques, the most relevant ones include dynamic light scattering (DLS) equipment that uses automatic sampling and dilution systems^7–9^ or fiber-optic DLS,^10^ automatic continuous online polymerization ACOMP,^11,12^ and fractionation methods^13–15^ like field-flow fractionation techniques (e.g., asymmetric-flow, AF4, and sedimentation-flow, SdFFF), and capillary hydrodynamic fractionation chromatography (CHDF). Turbidity measurements have also been used to online monitor PSD^16–18^ and particle size^19,20^ in emulsion polymerization reactions at lab scale.

The development of in-line PSD measurement equipment is more interesting than online ones because if appropriate sensors were available, there should not be a necessity to sample or dilute the sample. However, this sample preparation requirement makes it substantially more difficult to develop such an instrument. Indeed, Zhang et al.^6^ emphasized in their review that “*at present (2021) it is not feasible to determine the particle size distribution of the latex produced by emulsion polymerization using in-line technology*”. In-line equipment to measure average particle size during emulsion, miniemulsion, suspension, and dispersion polymerizations based on spectroscopic techniques has been reported. Raman spectroscopy,^21,22^ but more commonly NIR spectroscopy,^23–29^ has been used to illustrate that spectra taken in-line by means of a probe inserted in the emulsion polymerization reactor are affected by the particle size of the latex (in some particular region of the spectrum), and hence the average particle size can be retrieved by extensive multivariate calibration; in other words, the sensor is not a universal technique to measure particle size because calibration must be performed for each specific emulsion system. In the same way, very recently, Rust and Pauer^30^ reported the monitoring of a concentrated emulsion polymerization of VAc/VeoVa^31,32^ using in-line turbidity measurements (by means of nephelometry probes) and a calibration for the particular copolymer case analyzed. As for the other spectroscopic techniques, although the technology is robust, it is not universal and requires building calibration models for each polymerization system.

In addition, other scattering techniques have also been reported as in-line sensors to measure particle size in emulsion polymerization processes. In some cases, the measurement cell has been used or modified to be used as the polymerization reactor, and hence very diluted reactions were only tested but cannot be used to monitor commercial-like emulsion polymerizations.^33,34^ An interesting novel in-line DLS probe was developed by de Kanter et al.^35^ to monitor microgel production. The probe is composed of a custom-designed head probe that is mounted in a commercial fiber-optical DLS probe. The whole ensemble can be immersed in the reaction medium and hence allows monitoring polymerizations with up to 40 wt % solids content. The task of this probe head is to separate a small amount of volume from the bulk fluid, which can then be measured using the commercial probe. The probe was tested in the synthesis of microgels in a 1L reactor under very diluted conditions (1.5 wt %), and hence it remains to be demonstrated the robustness of such an in-line sensor for typical emulsion polymerization formulations. Synchrotron Small Angle X-ray scattering, SAXS, has also shown potential to in-line measure average particle size in emulsion and miniemulsion polymerization^36–38^ using specially designed reactors with an agitated vessel connected to a capillary where the SAXS measurement is carried out. As for the other scattering equipment, its implementation in larger laboratory reactors is not possible.

Among the in-line technologies to measure particle size, Photon Density Wave Spectroscopy (PDWS) is likely the most robust and interesting one to monitor high solids content emulsion polymerization processes. PDWS is based on photon transport theory (incorporating multiple scattering), Mie theory, and the theory of time-dependent light scattering determines the absorption and scattering properties of highly turbid samples, which allows for a dilution-free particle size determination by introducing a probe directly into nondiluted samples.^39–44^ Contrary to the other in-line techniques described above, PDWS does not require any calibration, although to correctly obtain the particle sizes of the dispersion, some parameters (e.g., refractive index of the disperse phase and concentration of the dispersed phase) are needed in-line to transform the reduced scattering coefficient into accurate particle size. This condition makes the use of this equipment challenging for real-time precise data acquisition.^40,41,45–49^

In this work, a novel online sensor to analyze particle size distribution is presented: the Optofluidic Force Induction (OF2i) technology.^48–50^ OF2i is a counting method based on the “optical tweezers” principle pioneered by Ashkin.^51^ OF2i takes these several steps further: Within a continuously working measurement cell, it focuses a donut-shaped laser (vortex beam) through the liquid sample to exert optical force on the particles that pushes them forward and turns them around the beam’s center. Nanoparticles become optically trapped and move along their spiral trajectories, which minimizes interparticle collisions that would otherwise disrupt measurement and makes it possible to follow the particles as they move through a measurement cell. The scattered light is recorded by an ultramicroscope located perpendicular to the direction of the flow and allows detecting the trajectories of the particles and hence particle velocities.^48^ The speed of their movement correlates with their particle size.^48–50^

The OF2i equipment was coupled with an automatic dilution system to test the suitability of the technique to real-time monitor the PSD during batch and semibatch emulsion polymerization processes of different monomer systems (e.g., homopolymerization of styrene and copolymerization of all (meth)­acrylic comonomers). All the results were compared to the results obtained offline by DLS and CHDF techniques, both used as validation technologies, as well as Transmission Electron Microscopy (TEM), to validate PSD results.

## 2 Experimental Section

### 2.1 Materials

Monomers were used as received: styrene (S, Scharlau, 99% purity), methyl methacrylate (MMA, Thermo Fisher Scientific, 99% purity), *n*-butyl acrylate (BA, Thermo Fisher Scientific, 99% purity), and methacrylic acid (MAA, Thermo Fisher Scientific, 99.5% purity). Sodium dodecyl sulfate (SDS, Aldrich, ∼100% purity) and Dowfax 2A1 (kindly supplied by Dow Chemical, a 45 wt % solution) were used as emulsifiers. Ammonium persulfate (APS, Aldrich, >98% purity) and sodium metabisulfite (*Na*_2_ *S*_2_ *O*_5_, Fluka, >98% purity) were used as initiators, and sodium bicarbonate (*NaHCO*_3_, Aldrich, >99.7% purity) as a buffer. As a continuous medium in the emulsion polymerizations and to dilute the samples for DLS analysis, deionized high-purity water obtained through a Merck Millipore system (DI-water) was used (water conductivity lower than 1 μS/cm).

### 2.2 Synthesis of Latexes Monitored by OF2i

OF2i was used to monitor in real-time the PSD in two different emulsion polymerization formulations: polystyrene latexes (S 100 wt %) and polyacrylate latexes (methyl methacrylate/butyl acrylate/methacrylic acid: MMA/BA/MAA 64/34/2 wt %). All reactions were carried out in a 0.5 L jacketed glass reactor equipped with a sampling device, a nitrogen inlet, a feeding inlet, a Pt-100 probe, the OF2i sampling tube, and a stainless steel anchor-type stirrer rotating at 150 rpm. The reaction temperature was 85 °C and it was controlled by a LAUDA ECO GOLD thermostatic bath. The flow rate of the monomer preemulsion in the semicontinuous experiments was controlled by a STEPDOS 08 RC pump.

Two polystyrene latex syntheses were monitored: an *ab initio* batch reaction (RB1) and a seeded semibatch emulsion polymerization reaction (RB4) aiming at producing a bimodal latex.

In the *ab initio* batch emulsion polymerization of styrene (RB1), the entire reaction was monitored by OF2i. For that, styrene, water, and emulsifier (below the critical micellar concentration, cmc, of the surfactant SDS) were added to the reactor. Then, the mixture was heated to the reaction temperature, and once the temperature was reached, the initiator solution was added as a shot. The emulsion polymerization was monitored for 4 h. The recipe for this reaction was taken from literature^52^ and can be found in Table S1.

For the monitoring of the synthesis of a bimodal PSD latex (RB4), first a PS seed (see formulation in Table S2) with an average particle size of 180 nm and 2.32% of solids content was loaded in the reactor together with the initiator, and the mixture was heated to 85 °C. Then, the seed latex was grown by feeding a monomer preemulsion for 180 min. After 100 min of starting the monomer feed, a shot of seed particles (the same amount of particles used in the reactor load) was introduced in the reactor to generate the bimodal latex, and the whole process was monitored in real-time by OF2i. The formulation of this reaction is presented in Table S3.

For the acrylate copolymer case (MMA/BA/MAA 64/34/2 wt %), two seeded semibatch emulsion polymerization reactions were performed to monitor the particle growth: in a first experiment (RB2), a seed latex of 55 nm particle size (see Table S2) was grown to a latex of 350 nm at a solids content of 40 wt %; in the second experiment (RB3), a seed latex of 200 nm particle size (see Table S2) was grown to a latex of 450 nm with a solids content of 20 wt %.

The formulations for the experiments RB2 and RB3 are presented in Table S3. Briefly, the selected seed plus an initiator solution were charged in the reactor and heated to the reaction temperature (85 °C). Then a preemulsion of the comonomer mixture was fed for 180 min, and after feeding the reaction, it was maintained for another 30 min at reaction temperature. The whole process was monitored by OF2i.

In all the reactions, samples were withdrawn every 15 min during the first 60 min of the reaction and every 30 min after that time to analyze the evolution of monomer conversion and particle size offline by DLS (all samples). In the bimodal latex case (reaction RB4), the same samples were also analyzed by CHDF, as well as the final latex samples of all reactions. The final samples were also analyzed by TEM.

A summary of the conditions of the reactions, including solids content (SC), amount of seed, and targeted particle diameters, is presented in Table 1. The dilution ratio and the refractive index (RI) used in each reaction for OF2i analysis are also shown in Table 1.

**Table 1:** Summary of Characteristics of the Reactions Performed to Test OF2i Technology

Reaction	Composition	SC seed/%	*d_N,DLS_* seed^a^/nm	SC targeted/%	*d*_*p*_ targeted/nm	Dil. Ratio 1:X	RI
RB1	S	-	-	9	-	2E5	1.59
RB2	MMA/BA/MAA:64/34/2	0.33	55	40	350	1E6	1.47
RB3	MMA/BA/MAA:64/34/2	4	200	20	450	2E5	1.47
RB4^b^	S (bimodal)	2.32	180	25	400/200	2E5	1.59

a Seed particle sizes are number-average particle sizes measured by DLS using the cumulants method.

b A shot of small particles is added after 100 min of feeding.

### 2.3 Optofluidic Force Induction

The OF2i equipment is composed of a laser source, a microfluidic cell from which the diluted sample is flowing with a continuous flow, and an analysis camera perpendicular to the flow direction. When the light is emitted in the same direction as the flow direction, for appropriately chosen laser power and flow velocities, a certain momentum is transferred from the laser beam to the particles, which then results in optical forces. These forces will make particles eventually be trapped on the laser intensity maximum in the measurement cell perpendicular to the flow (2D trapping). Furthermore, when the laser beam and the fluid propagate in the same direction (Figure 1a), the nanoparticles that are suspended in the fluid experience size-dependent velocity changes, which are observed and can be used to extract particle size and particle size distributions, their terminal velocity being a sum of the fluid velocity and the velocity due to the optical forces (Figure 1c). Moreover, the particles scatter the laser light, and part of the scattered light reaches the detection point of the camera (which is located perpendicular to the flow direction, as shown in Figure 1b), where the scattered light is recorded in real-time and continuously.^48–50^ The equipment is controlled by proprietary software called H.A.N.S.

**Figure 1:**
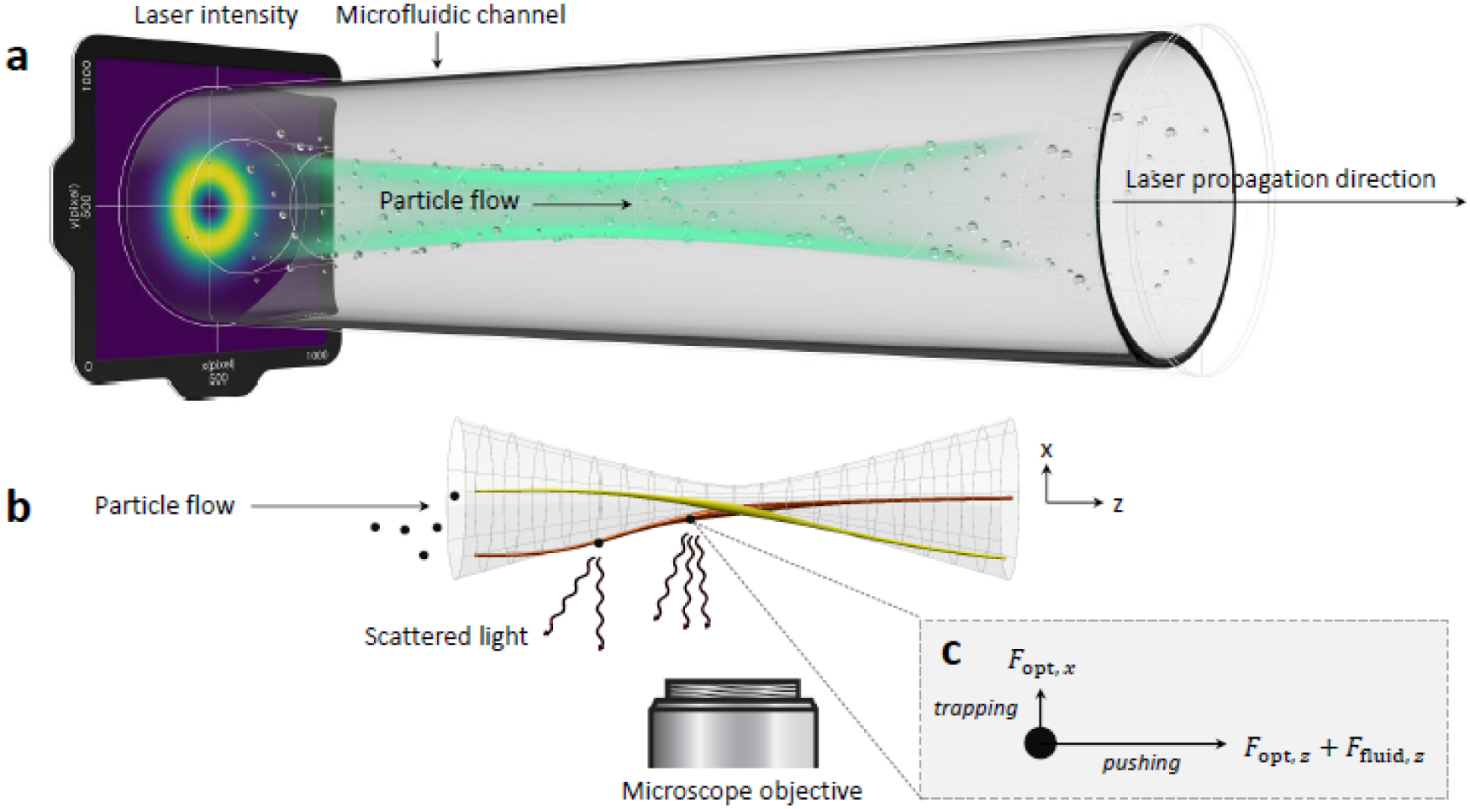
Working principle of the OF2i scheme. (a) Nanoparticles in suspension are pumped through a microfluidic flow channel at a constant flow rate alongside a weakly focused vortex beam. (b) Particles sufficiently close to the intensity maximum (gray cone) become optically trapped in radial direction by gradient forces (c, trapping). Trapped particles experience size-, shape-, and material-dependent scattering forces (c, pushing), leading to velocity enhancements in laser propagation direction. The light scattered off the particles is detected using an ultramicroscope setup, which allows monitoring of velocity changes of individual particles along the flow channel. Figure reproduced with permission from ref. 50. Copyright American Physical Society (2022).

The laser applies forces to the particles that are flowing through the cell. The purpose of this laser is 3-fold: first, the optical forces in the transverse directions (*x*, *y*) push the nanoparticles to the intensity maxima of the laser field, such that particles propagating sufficiently close to the maxima become optically trapped in the transverse directions; second, the optical forces in the laser propagation direction (*z*) push the particles and lead to velocity changes depending on size and material properties (RI and form); third, the particles scatter light, which can be monitored outside the cell.^50^

The velocity of the particles will be the sum of the velocity of the fluid and the velocity produced due to the effect of the optical forces over the particles and can be defined by via Newton’s equation of motion (eq 1).

1 $$m\mathrm{z̈}=F_{opt}\left(z,R\right)+F_{drag}\left(R\right)+F_{brownian}$$

where *m* is the mass of the nanoparticle, which might include the added mass due to the fluid, *z̈* is the acceleration of the particles, *F_opt_* is the force that the laser exerts on the particles resulting in velocity enhancements; *z* and *R* are the position of the particle in the laser beam and the hydrodynamic radius, respectively, *F_drag_* is the drag force acting on the particle moving through the fluid, and *F_brownian_* accounts for the stochastic forces due to thermal fluctuations (also known as Brownian motion). For the force acting on a sphere moving through a viscous fluid (*Re* ≪ 1) with velocity *v* we consider Stokes’ drag (eq 2),

2 $$F_{drag}\left(R\right)=-6\pi \mu R\left(v-v_{fluid}\right)$$

where *v* is the velocity with which the particles are moving, *v_fluid_* and *μ* are the velocity and dynamic viscosity of the fluid, respectively. In deriving eq 2, we have assumed a capillary with an inner diameter of *d_cap_* ≈ 1 mm as well as flow rates in the range of single-digit μL/min, which is representative for most OF2i applications. In our experiments, we set the flow rate to a value of approximately 4 μL/min, which corresponds to a fluid velocity of *v_fluid_* ≈ 0.1 mm/s and fulfills the condition of a laminar flow.

For sufficiently large spheres, say for diameters above 10 nm, the momentum relaxation time is so short (the terminal velocity is reached fast) that it can be approximated as *v̇* ≈ 0 *(mz̈* ≈ 0). Also, the Brownian motion does not play a decisive role for large spheres, and it is negligible in comparison to the flow speed (*F_brownian_* ≈ 0). Thus, for the condition that the optical force is balanced by the drag force (*F_opt_* (*z*,*R*) = −*F_drag_* (*R*) = 6πμ*R*(*v* – *v_flui__d_*)), the following definition of particle velocity is obtained (eq 3). Thus, knowing the velocity of the particle, its radius can be retrieved.

3 $$v\left(z,R\right)=v_{fluid}+\frac{F_{opt}\left(z,R\right)}{6\pi \mu R}$$

This parameter-free model relates the velocity of a nanoparticle *v*(*z*, *R*) to the optofluidic forces present in the microfluidic channel. In the next step, we compute for a given particle refractive index (see Table 1, RI) the particle velocity in the focal region of the beam for a specified particle size range. This provides us with a lookup table that relates the simulated particle velocities to the corresponding sizes and allows for the determination of a PSD. For a detailed explanation of our simulation approach and the underlying working equations, we refer the reader to refs. 48 and 52, where we discuss all model ingredients (e.g., beam diameter, flow rates, laser power) in great detail.

Depending on the analyzed material and particle size, three different behaviors can be observed when flowing a dispersed medium through a cell where a laser is focused in the direction of the flow: a) particles are trapped in the laser since the beginning and they are moved through the laser because of the just introduced forces; b) particles are not trapped since the beginning, but they are attracted to the maximum intensity point of the beam due to the optical forces, becoming trapped finally (the smaller the particle, the bigger the force needed to trap it and take it to the maximum intensity point); c) particles are not trapped at any moment, due to being too far from the intensity maxima. The particles observed by OF2i are from the first two types, while the third type cannot be observed.^48^ Thus, particles sufficiently close to the laser beam become optically trapped in the radial direction (gradient forces) and experience velocity changes in the propagation direction of the laser beam. The minimum size for trapping (and therefore, for being in each of the scenarios) depends on the laser power and the numerical aperture of the focusing lens, the configuration of the microfluidic cell, and the refractive index (*n*) of the analyzed material.^48^

Therefore, when inferring the PSD from OF2i measurements, one has to take into account the fact that larger particles become trapped more easily than smaller ones, owing to the increase of optical forces with increasing particle size. To try to compensate for this bias in the obtained results, a parameter called active volume is considered (eq 4).^50^

4 $$V_{active}\left(d,n_{p}\right)=\left[\pi r_{cut}^{2}\left(d,n_{p}\right)\right]v_{fluid}t_{meas}$$

where the term in brackets is the cross-section in the transverse direction, *r_cut_* is the cutoff radius with respect to the center of the laser beam where a particle of diameter *d* and refractive index *n_p_* still becomes trapped, *v_fluid_t_meas_* is the sampling distance spanned along the propagation direction of the flow.

Thus, the probability for a particle to be trapped in the laser changes depending on its size. The larger the particle size, the higher the probability for the particle to be trapped in the laser and thus to be detected during the measurement. So, the active volume compensates for this fact, correcting for the percentage of each particle size that is trapped $\left(N_{p}\propto \frac{N_{p,detected}}{V_{active}}\right)$ . Because each particle is measured individually as it flows through the measurement cell, the PSDs obtained through OF2i are on a number basis.

In OF2i, the applicable size range is governed by both physical principles and the material properties of the particles under investigation. For particles that are small compared to the wavelength of the exciting laser (<100 nm), the scattering forces in the *z*-direction scale with the particle diameter *d* as *F_scat_* ∝ *d* ^6^. This size dependence results in insignificant velocity enhancements for particles with a low refractive index.^48,50^ For materials with a high refractive index, however, the scattering forces might suffice for a velocity-based analysis of particle size, thus posing a sample-dependent lower size limit. BRAVE is currently implementing and testing a correlative measurement approach that combines particle trajectory information with static light scattering. In the future, this might allow us to overcome the difficulties in analyzing small particles based on their velocity.

The “Measurement validity “retrieves the percentage of particles analyzed that are in the sizing range of the OF2i equipment used (which is 100 nm to 3000 nm). Moreover, it gives information about the percentage of the counted particles laying in the just mentioned areas. As an example, a validity of 97% (2%/1%) means that 97% of the particles are in the sizing range of 100 nm to 3000 nm, 2% of the particles are below 100 nm (particles are tracked but too small for accurate sizing) and 1% of the particles are above 3000 nm (particles are tracked but too large for accurate sizing).

For the particles in the sizing range with known active volume (calculated from the particle size) and particle count, the concentration can be calculated, and hence the particle size distribution. A deeper insight into the theory and working principle of OF2i technology can be found elsewhere.^48–50,53^

Commercial emulsion polymerization processes produce latexes with solids contents in the range of 35–60 wt %. Therefore, to monitor these processes with the OF2i, a dilution system is needed because the OF2i equipment as a counting method is set to directly analyze dispersions with concentrations up to 10^10^ objects/mL. The OF2i instrument used in this work comprised a built-in dilution system.

The automated dilution system consists of two stages and is designed to cover a wide range of dilution ratios within short process times. In stage 1, the raw sample is delivered using a multiroller peristaltic pump, ideal for very low flow rates, while the dilution media is added via a displacement pump based on an innovative design using a peristaltic principle. A static mixer ensures homogeneous mixing.

Stage 2 operates in a pulsed flow mode with alternating loading and mixing cycles to achieve highly efficient high-ratio dilutions. A precise multipiston pump handles the prediluted sample, while a microgear pump supplies the dilution media. Combined with low-dead-volume microfluidic components, this enables a compact design, stable flow profiles, and reliable operation even at very low flow rates. A microfluidic mixer based on multilamination is used for homogenization in stage 2. Both stages incorporate volumetric flow sensors based on the differential pressure principle to ensure precise flow control.

The dilution system can achieve dilution ratios ranging from 1:10^2^ to 1:5 × 10^6^. However, total uncertainty increases significantly at very low dilution ratios; below 1:3 × 10^2^. To optimize performance, the total range is divided into several smaller working ranges, each with predefined parameter sets. Within each range, the flow rates of the dilution media path and the raw sample path are maintained at constant values. Different dilution ratios within the active working range are achieved by precisely controlling the flow rate of the single-diluted sample in stage 2. The effect of different dilution ratios on the measured particle size was analyzed, and it was found that in the range of 1:10^3^ to 1:2 × 10^5^ the measured average particle size was not affected (see Supporting Information for additional details).

To enable automated sample preparation and subsequent OF2i measurements, dedicated software was developed, which can also be controlled from the main OF2i software, H.A.N.S. By setting a dilution ratio in the H.A.N.S. software, the operating parameters are automatically calculated, and all dilution steps are executed. Once the sample preparation is complete, the diluted sample is automatically transferred to the OF2i device and measured.

### 2.4 Dynamic Light Scattering

A Zetasizer Nano Series (Malvern Instruments) DLS device was used for reference measurements in this work. For the analysis, the latex withdrawn from the reactor was diluted with DI-water until an attenuation number of 6–7 was achieved. Measurements were taken 4.65 mm above the bottom of the cuvette and carried out at room temperature with an equilibration time of 120 s. Three repeated measurements, each consisting of 13 subruns, were conducted for each sample. All reference particle diameters shown in the results section are number-average particle diameters. The RI used for the dispersed (polymer) and continuous phase (water) was 1.47 for MMA/BA/MAA copolymer, 1.59 for polystyrene, 1.33 for water, and the viscosity of the continuous phase was considered to be 0.8872 cP at the measurement temperature.

### 2.5 Capillary Hydrodynamic Fractionation Chromatography

Particle size distributions were obtained offline by capillary hydrodynamic fractionation chromatography, CHDF. A CHDF3000 instrument (Matec Applied Sciences) was used for the analysis, with its proprietary CHDF3000 PDA 2.1 software and C-204 column (internal diameter 20 μm). The CHDF was operated under the following conditions: A flow rate of 1.4 mL/min, a temperature of 35 °C, the detector wavelength at 200 nm, and a sample concentration of 0.05 wt %. Samples were injected into the instrument, and the analysis time was around 15 min. The samples were analyzed using an UV absorbing extinction file corresponding to the PS polymer that is optimized in the instrument. The refractive index of polystyrene was used to obtain the concentration for each particle population (real = 1.871 and imaginary = 0.525).

### 2.6 Transmission Electron Microscopy (TEM)

Transmission electron microscopy, TEM, was also used to analyze offline the particle size distribution. A Tecnai G2 20 Twin device operating at an accelerating voltage of 200 keV was used for the analysis. A drop of sample (SC 0.4 wt %) was deposited in a carbon-coated standard Cu TEM grid and dried at 5 °C in a fridge. ImageJ software was used to analyze the PSD, counting a minimum amount of 500 particles per latex.

## 3 Results and Discussion

OF2i was assessed to monitor in real-time four emulsion polymerization reactions, RB1-RB4. RB1 is an *ab initio* batch emulsion polymerization of styrene; RB2 and RB3 are two seeded semibatch emulsion polymerizations of a (meth)­acrylic copolymer where particle growth in a broad size range is monitored, and RB4 is a seeded semibatch emulsion polymerization of styrene seeking the production of a bimodal latex. Instantaneous and overall conversion data of all reactions can be found in Figure S1.

### 3.1 *Ab Initio* Batch Emulsion Polymerization of Styrene (RB1)

As discussed in the Introduction and in Section 2.3, the lower limit of detection of the OF2i equipment used is around 100 nm. Therefore, the classical emulsion polymerization of styrene in the presence of micelles (heterogeneous or micellar nucleation) cannot be monitored by the OF2i because too small particles are produced. Thus, the *ab initio* emulsion polymerization of styrene was carried out in absence of micelles (i.e., with SDS concentration below the cmc). This yields relatively large polymer particles from the beginning of the polymerization process, because of the lack of micelles and the low surface charge density that favors aggregation of the particles.^54^ The results obtained by OF2i during monitoring of the RB1 emulsion polymerization reaction are presented in Figure 2.

**Figure 2:**
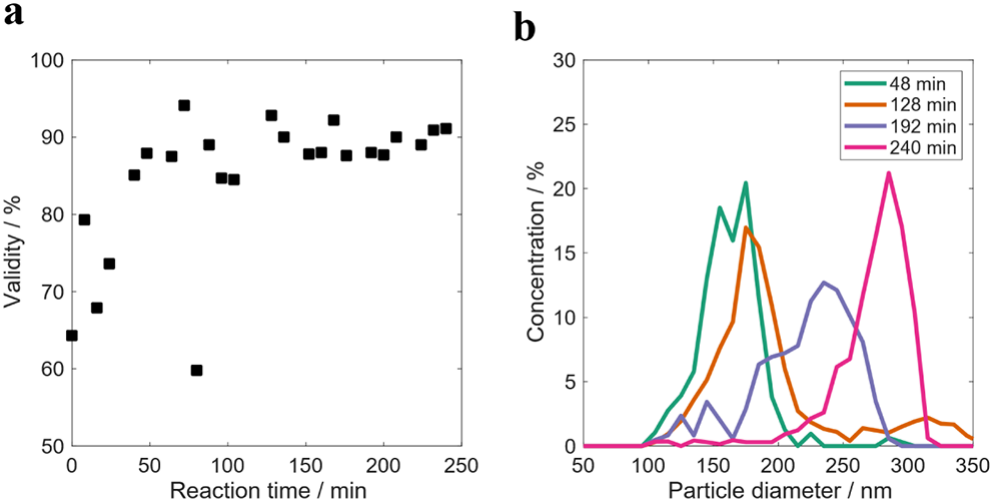
OF2i results obtained during the RB1 reaction. a) Validity of OF2i results and b) number-based PSD evolution.

As depicted in Figure 2a, the validity (portion of particles sized vs portion of particles detected) of the OF2i results was lower than 80% for the first 40 min of the reaction and then approached to 90%. Once the particles are big enough to be accelerated to reach velocities that are higher than the velocity of the OF2i flow, the validity is higher, being close to 90%. Figure 2b shows the evolution of the PSD during the reaction, showing a broadening of the distribution and a shift to higher values with reaction time. The PSD at 48 min is unimodal with a number-average particle size of 160 nm. At 128 min the PSD is bimodal with a second population, formed by aggregation, in the range 250–350 nm. As the reaction proceeds, a larger and broader PSD skewed to small particles (192 and 240 min) is measured. This evolution is in agreement with a poor stability of the latex because of the low surfactant concentration employed and a homogeneous particle nucleation process followed by aggregation to reach stable particle sizes with sufficient surface coverage of the emulsifier.

Figure 3 shows the time-evolution of the number-average particle size during the reaction and the comparison of the PSD of the final product with other offline measuring techniques. Figure 3a shows that the particle size of the dispersed polystyrene particles is increasing during the entire process, which was adequately monitored by OF2i. The same trend was observed with offline DLS analysis, showing good agreement of results for most of the reaction. The difference can only be seen for the first 40 min of the reaction, where the particle size measured by DLS is smaller than the values retrieved from OF2i. This is attributed to the limitation of the actual OF2i equipment to accelerate the small particles to higher velocities than the flow velocity, yielding average values higher than the actual ones. This is supported by the validity of the OF2i results (see Figure 2a).

**Figure 3:**
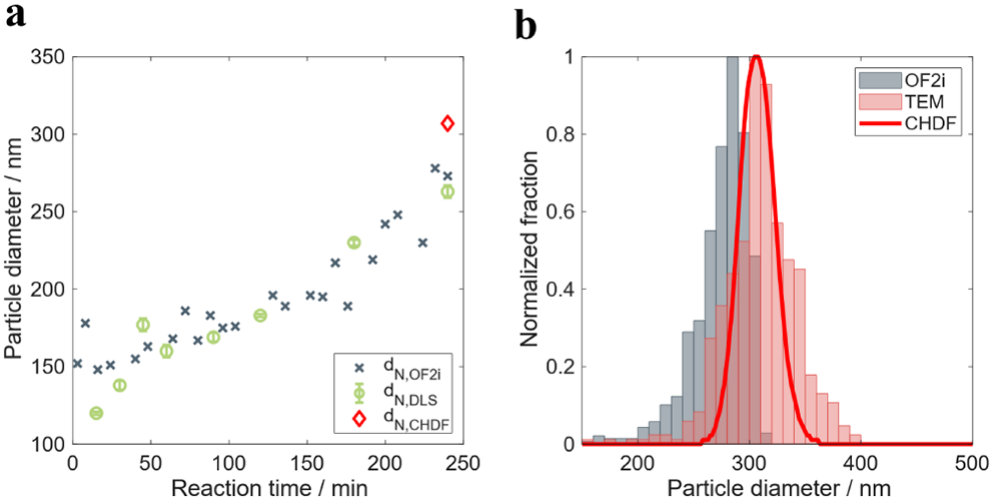
OF2i results obtained during the RB1 reaction. a) Evolution of the number-average particle size during the RB1 reaction, and b) number-based PSD of the final latex compared to CHDF and TEM results.

Regarding the constant increase in particle size with reaction time, this can be attributed to the low concentration of emulsifier (below cmc of SDS) that makes the polymer particles formed by homogeneous nucleation unstable. The increase in the particle size is an indication of continuous nucleation and aggregation processes occurring simultaneously in the reaction.

The PSD of the final latex was also compared to the PSD obtained by CHDF and TEM (Figure 3b). The comparison shows differences between offline and online techniques. While CHDF and TEM PSDs overlap with broader distribution measured by the TEM, the PSD retrieved from the OF2i, although as broad as that obtained by TEM, slightly shifted to lower sizes.

We estimate that this deviation might be introduced by two simplifications made in our theoretical framework and corresponding evaluation algorithms. In our current implementation, we set the hydrodynamic radius of a particle, *R*, equal to the radius of a hard sphere entering the optical force calculation. In general, these radii might differ, depending on the sample under investigation.^48^ In OF2i, the incoming vortex beam is generated through a q-plate, and the fields are usually described by Laguerre-Gaussian (LG) modes. However, vortex beams generated by q-plates are more adequately described by the family of Hypergeometric-Gaussian modes,^55^ which differ in their divergence behavior and intensity distribution compared to LG modes. Both the assumptions regarding the hydrodynamic radius and the description of the incoming beam play a decisive role in the extraction of particle sizes and can explain the deviation in the resulting PSD. In future work, these implementations might provide a more accurate picture of particle dynamics and PSDs.

### 3.2 Seeded Semibatch Emulsion Copolymerization of MMA/BA/MAA (RB2 and RB3)

Experiment 1. Growing seed particles from 55 to 350 nm (RB2)

The reaction started from a seed latex with 55 nm of particle size and a solids content of 0.33% and the final particle size targeted was 350 nm with a solids content of 40 wt %.

Figure 4 shows the validity (Figure 4a) and the PSD (Figure 4b) evolution measured online by OF2i during the growth of the seed latex in the copolymerization reaction RB2.

**Figure 4:**
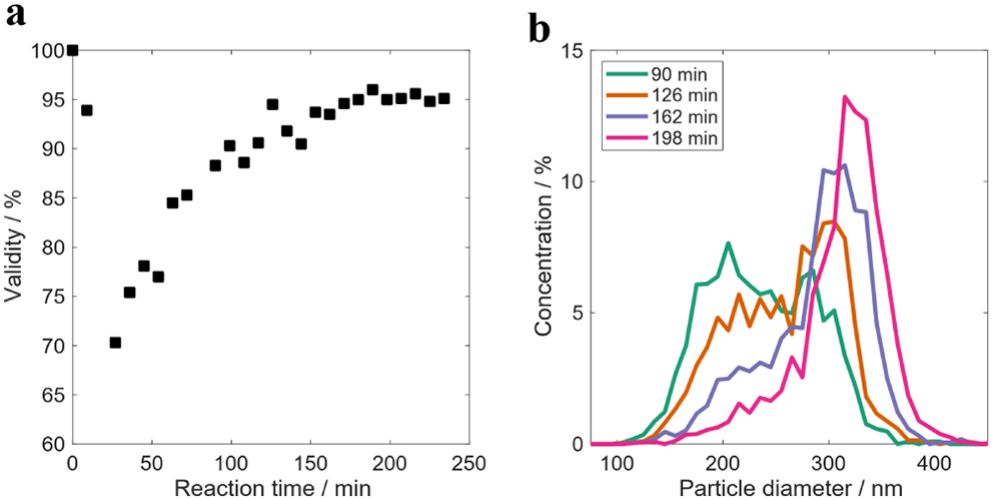
OF2i results from experiment RB2: a) validity of the results of the OF2i, and b) number-based PSD measured online by OF2i during particle growth.

Figure 4a shows that validity was very low during almost the first 2 h of reaction (validity lower than 90%), which means that a large fraction of the particles detected by the equipment could not be characterized properly because their size was too small to be accelerated enough and hence their size correctly determined. Figure 4b presents the PSD measured at different reaction times, for the times where the validity is close to or higher than 90%. The evolution of the PSD differs from the typical evolution of this type of process,^56^ where unimodal PSD is shifted to larger sizes with certain broadening during the polymerization because of different volumetric growth rates of the particles. Although in Figure 4b we can also observe a shift to large sizes (especially the second mode of the distribution), we observed a bimodal PSD with a low mode in the range 150–250 nm. This mode gradually disappears as the reaction proceeds. This evolution is likely an artifact coming from the difficulties to measure small particles (50–150 nm range) and hence the obtained PSD is biased. This is supported by the low validity numbers obtained up to 2 h of reaction.

Figure 5a presents the number-average particle size evolution calculated from the PSD’s and the comparison with the same average obtained from offline measurements with DLS. As can be seen, the average value calculated by OF2i up to 100 min of reaction overestimates the values measured by DLS, but beyond this time both results overlap very well. This is in very good agreement with the validity values; in other words, when the validity values are above 90%, the reliability of the OF2i increases. As discussed before, the low validity values are a consequence of the current equipment configuration that limits the detectable particle size of this copolymer with relatively low refractive index to values of about 200 nm.

**Figure 5:**
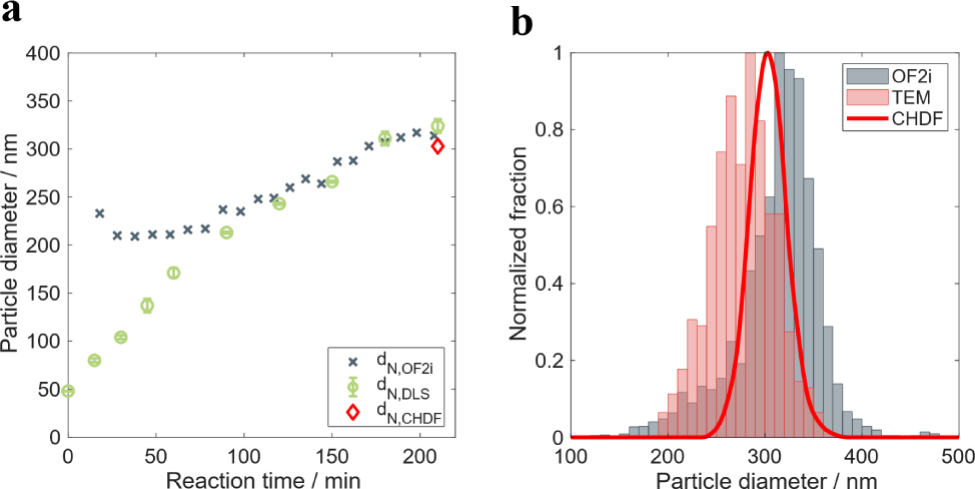
a) Number-average particle size evolution measured online by OF2i and offline by DLS during particle growth experiment RB2; b) number-based PSD of the final sample of the latex RB2 measured online by OF2i and offline by CHDF.

The comparison of the PSD measured by OF2i with respect to CHDF and TEM of the final product of RB2 latex is presented in Figure 5b. OF2i and TEM PSD’s are broader than that measured by CHDF. Although there is substantial overlapping between the three techniques, the TEM PSD is shifted to smaller sizes (the fraction of particles below 250 nm is larger in TEM) and OF2i PSD is shifted to larger sizes (and the fraction of large particles is higher than in CHDF and TEM).

Experiment 2. Growing seed particles from 200 to 450 nm (RB3)

Reaction RB3 aims to produce a latex with an average particle size of 450 nm and a SC of 20 wt %, starting from a seed with a particle size of 200 nm and a SC of 4%. Figure 6 presents the validity values and the PSD evolution along the reaction.

**Figure 6:**
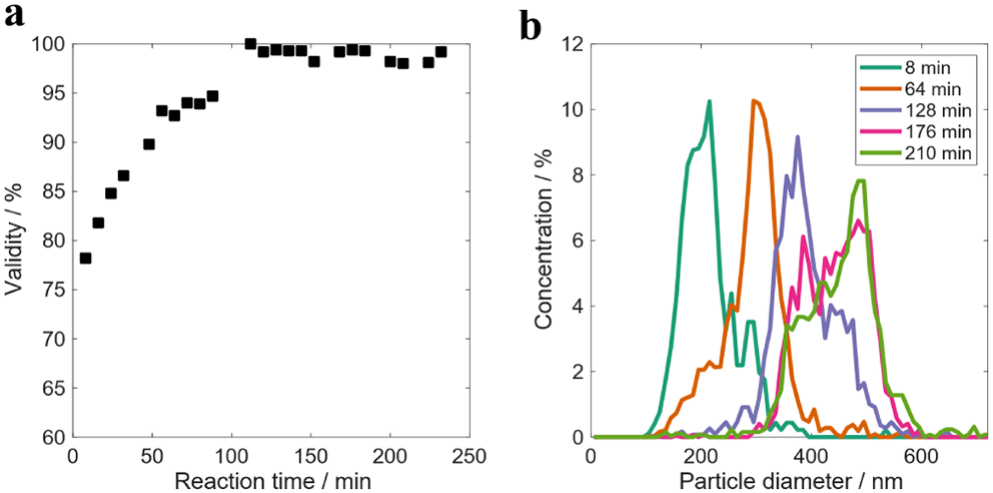
a) Validity of the results of the OF2i and b) number-based PSD measured online by OF2i during the particle growth experiment RB3.

In this experiment, the particle size of the seed was close to the lower limit detected in experiment RB2, so it was expected that OF2i would be able to monitor most of the reaction online. Indeed, the validity values presented in Figure 6a show that after the first 45 min (where values were between 80 and 90%), the validity was higher than 90%. Figure 6b presents the online PSD evolution. Particle growth is well captured by the shift in the maximum of the PSD, but the distributions show shoulders at lower and larger sizes than the maximum. One explanation for this observation can be the fact that the likelihood of trapping particles by OF2i is higher for larger particles (see references [50–52]), as mentioned above. Although a correction factor is used by OF2i to account for this, the correction used might not be completely accurate yet, and this introduces distortions in the PSD’s.

Figure 7a shows that both online and offline measurements overlap almost during the entire reaction. During the first 40 min, the OF2i values are slightly larger, in agreement with the lower validity values, because the particle size is close to the lower detection limit of the equipment. From this point on, both techniques provided the same average values up to the end of the feeding time. The last samples measured by OF2i are larger than those measured by DLS and CHDF. This discrepancy is also illustrated in Figure 7b, where the PSD of the final sample is plotted as measured by OF2i, CHDF, and TEM. Both offline techniques show smaller particle sizes than the OF2i, even if TEM measures a higher fraction of smaller particles than CHDF as in experiment RB2. But the OF2i PSD presents a fraction of particles at larger sizes, 450–600 nm, that are not present in the other techniques. We think that this discrepancy should be an artifact of OF2i; most likely some aggregation occurred in the dilution system.

**Figure 7:**
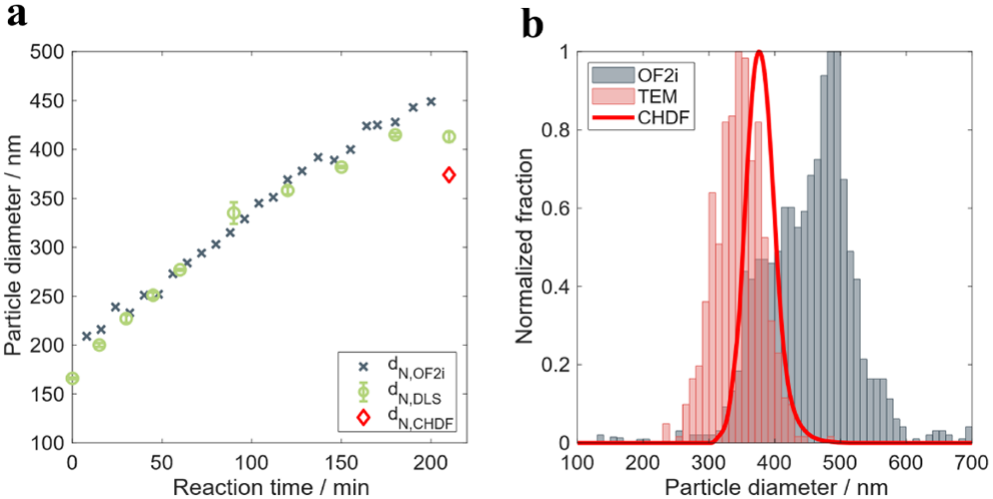
a) Time evolution of the number-average particle size measured online by OF2i and offline by DLS during the particle growth experiment RB3, and b) number-based PSD of the final sample of the latex RB3 measured online by OF2i and offline by CHDF and TEM.

### 3.3 Seeded Semibatch Emulsion Polymerization of Styrene to Produce a Bimodal Latex (RB4)

This section evaluates the suitability of OF2i to detect secondary nucleations that might result in bimodal PSD during emulsion polymerization reactions. Secondary nucleation is sometimes desired to generate a bimodal latex because this allows increasing solids content with little or no impact on the viscosity of the dispersion.^57^ However, other times secondary nucleations are not desired and may indicate a deviation of the targeted growth of the particles originating from a failure in the feeding of surfactant, initiator, or even monomer. In any case, early detection of the formation of a new crop of polymer particles during an emulsion polymerization process is desired.

Reaction RB4 was carried out to mimic the generation of a new population of small particles (large enough to be detected by OF2i) during a seeded semibatch emulsion polymerization of styrene. This reaction started with a seed latex with a particle size of 180 nm (slightly above the lower detectable limit by OF2i for polystyrene particles), and a particle size of 425 nm was targeted for these particles. However, at an intermediate point during the feeding of the monomer (after 100 min), a certain amount of seed latex (see formulation in the Supporting Information) was injected into the reactor in the same amount as in the beginning to create the bimodal latex. The recipe for the experiment can be found in the Table S3, and the evolution of the instantaneous and global conversions can be found in Figure S1.

OF2i was used to monitor the PSD online during the emulsion polymerization reaction. Furthermore, the samples were also analyzed by CHDF, as bimodal or multimodal populations are better detected by this chromatographic technique than with DLS.^58^ The final sample was also analyzed by TEM.

Figure 8 displays a comparison of the time-evolution of the PSD analyzed by OF2i and CHDF for the bimodal latex synthesis. Interestingly, the seeded semibatch emulsion polymerization resulted in a challenging experiment to assess the online performance of the OF2i in comparison with offline CHDF measurements. This challenge came from the fact that the reaction formulation led to the formation of a bimodal latex by the creation of small particles before the intended addition of the shot of the seed particles. Thus, at the beginning of the reaction, a seed with a monomodal particle size distribution was loaded into the reactor. Notably, the PSD measured by OF2i and CHDF mostly overlapped. At 60 min (before the shot addition of the seed), a bimodal PSD was already measured by OF2i, which was not expected. To understand this, it is worth noting that the seed was formed in a batch emulsion polymerization synthesis with the surfactant concentration below the cmc ([SDS] = 1.65 mM and cmc_SDS_ = 8 mM), and the surfactant feeding in the semibatch process was done in a higher proportion than its cmc ([Dowfax] = 1.65 mM and cmc_Dowfax_ = 0.125 mM), creating new particles by heterogeneous nucleation and producing a bimodal latex. CHDF also captures this new crop of particles and the growth of both populations as shown in the comparison at 60 and 90 min of reaction. After the addition of the seed particles (after 100 min of reaction), the concentration of the small population increases (the size of the added seed particles and formed *in situ* in the reaction are similar, and they are only seen as a single peak). OF2i captures well the increase of the population centered around 180 nm. The offline PSD measured by CHDF at this time matches well with OF2i. Beyond this point, both populations continue growing because of monomer addition, and this growth is well captured by OF2i that agrees relatively well with the offline CHDF measurement. Interestingly, at 210 min of reaction (after the postpolymerization step), the OF2i PSD presents a third low particle size mode (100–150 nm) population, which is also detected by CHDF. This new population likely originated from further micellar nucleation that took place due to the large amount of micelles in the preemulsion feed of monomer.

**Figure 8:**
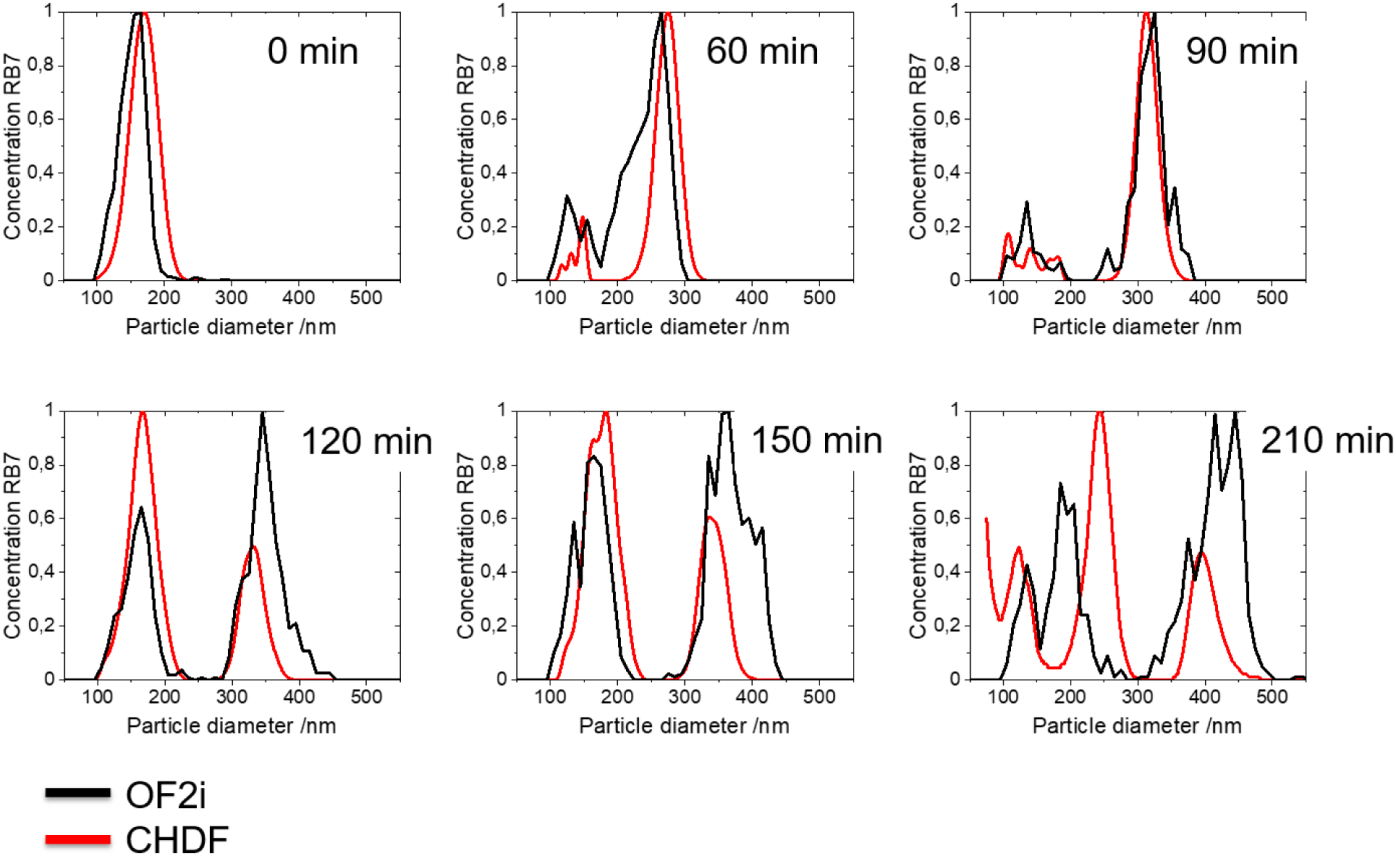
Number-based PSD evolution during the reaction RB4 measured online by OF2i and offline by CHDF.

To shed light on the trimodality of the PSD, the final sample was also analyzed by TEM. Figure 9 shows two representative micrograph images of the final latex as well as the PSD measured by three techniques: OF2i (online), CHDF, and TEM (offline).

**Figure 9:**
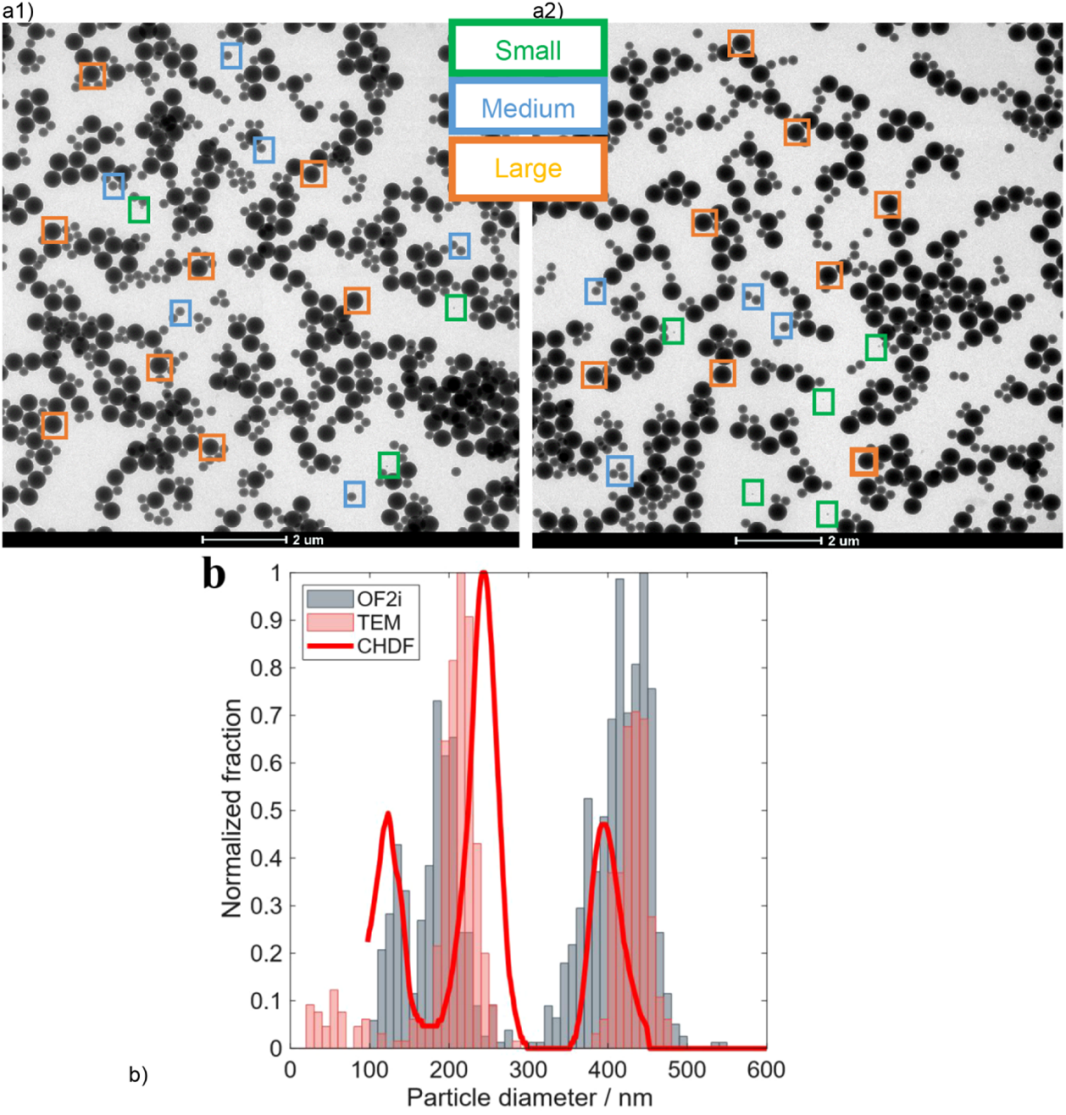
a1,a2) TEM micrograph images of the final latex of the RB4 reaction. b) Comparison of the number-based PSD obtained by OF2i (gray), CHDF (red), and TEM (light red) for the final latex of the RB4 reaction.

Figure 9a shows that three different populations can be distinguished by TEM in the final latex of the RB4 reaction in agreement with OF2i and CHDF measurements. Figure 9b displays the comparison of the PSDs obtained by CHDF, OF2i, and TEM. The three techniques are in relatively good agreement because the three populations are revealed in the different techniques, confirming the trimodality of the latex produced. Note, however, that the relative percentages and the ranges of each population are not in complete agreement. This difference comes from the fact that two of the methods are particle counting methods (OF2i and TEM), and the other one, CHDF, is a fractionation method that uses a UV spectroscopy detector and calibration to determine the particle size and PSDs. In the PSDs of Figure 9b, OF2i used 800 particles and TEM around 500 to determine the distributions, which also makes a difference in the obtained plots. However, most importantly, the three analytical techniques are able to detect the true PSD, and hence this reinforces the suitability of the OF2i technique as an online real-time particle size distribution monitoring technique for emulsion polymerization processes.

## 4 Conclusions

This study demonstrates the capability of the Optofluidic Force Induction (OF2i) technology to provide real-time monitoring of particle size distribution (PSD) during emulsion polymerization reactions. OF2i enables continuous online characterization of PSD throughout the reaction trajectory, capturing distinct mechanistic stages including primary particle nucleation (in micelle-free *ab initio* systems), particle growth (in seeded semibatch processes), and secondary nucleation events (mimicked via sequential seed particle injections during growth). The system delivers accurate PSD measurements for particle sizes exceeding 180 nm in polystyrene latexes and 200 nm in (meth)­acrylated copolymer latexes, with a temporal resolution governed by the dilution interface, currently permitting sampling at 10 min intervals.

OF2i represents, to our knowledge, the only commercially available instrument capable of *in situ* PSD analysis in stirred tank reactors, integrating automatic dilution and requiring no composition-specific calibration. The PSD outputs and mean particle diameters obtained via OF2i are in good agreement with established offline techniques including DLS, CHDF and TEM. Further enhancements to the optical and fluidic design of the OF2i system are expected to extend its sensitivity toward sub-100 nm particle detection.

## Supplementary Material



## Notes

Recipes for the *ab initio* and seeded semibatch emulsion polymerizations monitored with OF2i; evolution of the instantaneous and overall conversions of the monitored experiments; effect of the dilution ratio on the particle sizes measured by OF2i (PDF)
