# Supplementary material for: Optofluidic Force Induction for Online Monitoring of Particle Size Distributions in Emulsion Polymerization Reactions
Source: ACS Polym Au. 2025 Oct 2;5(6):871–82. doi: 10.1021/acspolymersau.5c00061 (PMC12874152; doi:10.1021/acspolymersau.5c00061)
Supplement: Supplementary file 1 [file lg5c00061_si_001.pdf]

Supporting Information for:

Optofluidic Force Induction for Online Monitoring of Particle Size Distributions in Emulsion Polymerization Reactions

Authors: [1]Usue Olatz Aspiazu, [2,3,4]Marko Šimić, [2]Michael Schnur, [4]Ulrich Hohenester, [2,3]Christian Hill, [2,3]Doris Auer, [1]Maria Paulis, *[1]Jose Ramon Leiza *[1]

[1] POLYMAT, Kimika Aplikatua saila, Kimika Fakultatea, University of the Basque Country UPV/EHU, Joxe Mari Korta zentroa, 20018 Donostia-San Sebastián.

[2] BRAVE Analytics GmbH, Stiftingtalstraβe 14, 8010 Graz, Styria, Austria

[3] Gottfried Schatz Research Center, Division of Medical Physics and Biophysics, Medical University of Graz, Neue Stiftingtalstraβe 2, 8010 Graz, Styria, Austria

[4] Institute of Physics, University of Graz, Universitätsplatz 5, 8010 Graz, Styria, Austria

Recipes

In the following, the precise recipes described in the experimental section of the main document are presented. Table SI 1 shows the composition of the RB1 batch emulsion polymerization reactions. Table SI 2 shows the composition of the SB1 and SB2 seeds synthesis used to perform reaction with different material compositions and Table SI 3 shows the information about the RB2-RB6 seeded semibatch emulsion polymerization reactions.

Table SI 1. Recipe of the polystyrene latex synthesized by batch emulsion polymerization (reaction RB1).

Reagents	RB1 (Mass/g)
S	32.7
SDS	0.15 (1.11 mM)
Water	467
APS	0.46
$NaHCO_3$	0.48
$Na_2S_2O_5$	0.5

Table SI 2. Recipe of the seeds synthesized by batch (SB1 and SB2) and semibatch (SB3) emulsion polymerization.

	Reagents	Seed SB1 (Mass /g)	Seed SB2 (Mass /g)	Seed SB3 (Mass / g)
Initial charge	S	0	48.05	0
	MMA	156.56	0	0
	BA	156.56	0	0
	SDS	12.48	0.225 (1.65 mM)	0
	APS	2.72	0.43	0.49
	$NaHCO_3$	0	0.44	0
	$Na_2S_2O_5$	0	0.34	0
	Water	470.08	471	297.74
	Seed SB1	0	0	4.42
Feed	MMA	0	0	62.91
	BA	0	0	33.42
	MAA	0	0	1.97
	Dowfax 2A1 (45 %)	0	0	2.18
	Water feed	0	0	100.85

Table SI 3. Recipe of the seeded semibatch emulsion polymerization reactions carried out (RB2-RB6).

	Reagent	Reaction/Seed		
		RB2 / SB1	RB3 / SB3	RB4* / SB2
Initial charge (mass/g)	Seed	2.43	65.66	80.05
	Water	273.06	244.16	231.63
	APS	1	0.46	0.58
Feed (mass/g)	S	0	0	110
	MMA	127.38	56	0
	BA	67.67	29.75	0
	MAA	3.98	1.75	0
	Dowfax 2A1 (45 %)	4.42	1.94	1.65 (3.22mM)
	Water feed	25.49	106.25	70

* 80.05 g of seed latex are added after 100 minutes of starting the monomer feed to create a bimodal latex.

Evolution of instantaneous and overall conversions during semibatch reactions

All monomer conversions were measured by offline gravimetric analysis of the withdrawn samples. For that, 2 grams of the withdrawn sample were deposited in a capsule and dried for 24 h at 60 ºC. The instantaneous gravimetric conversion $X_{instant}$ as function of time t is defined as the mass of polymer produced $m_{Polymer}$ divided by the mass of monomers fed until the sampling time $m_{monomer\_fed}$ (Equation 1). The fraction of dry sample weight not coming from the polymer (initiator, surfactant and hydroquinone) is negligible. The overall conversion X_{overall} as function of t is $m_{Polymer}$ divided by the total amount of monomer in the formulation $m_{monomer\_total}$ (Equation 2).

$$X_{instant}(t) = \frac{m_{Polymer}(t)}{m_{monomer\_fed}(t)} \quad \text{(Equation 1)}$$

$$X_{overall}(t) = \frac{m_{Polymer}(t)}{m_{monomer\_total}} \quad \text{(Equation 2)}$$

The results obtained for the semibatch reactions are presented in Figure SI 1.

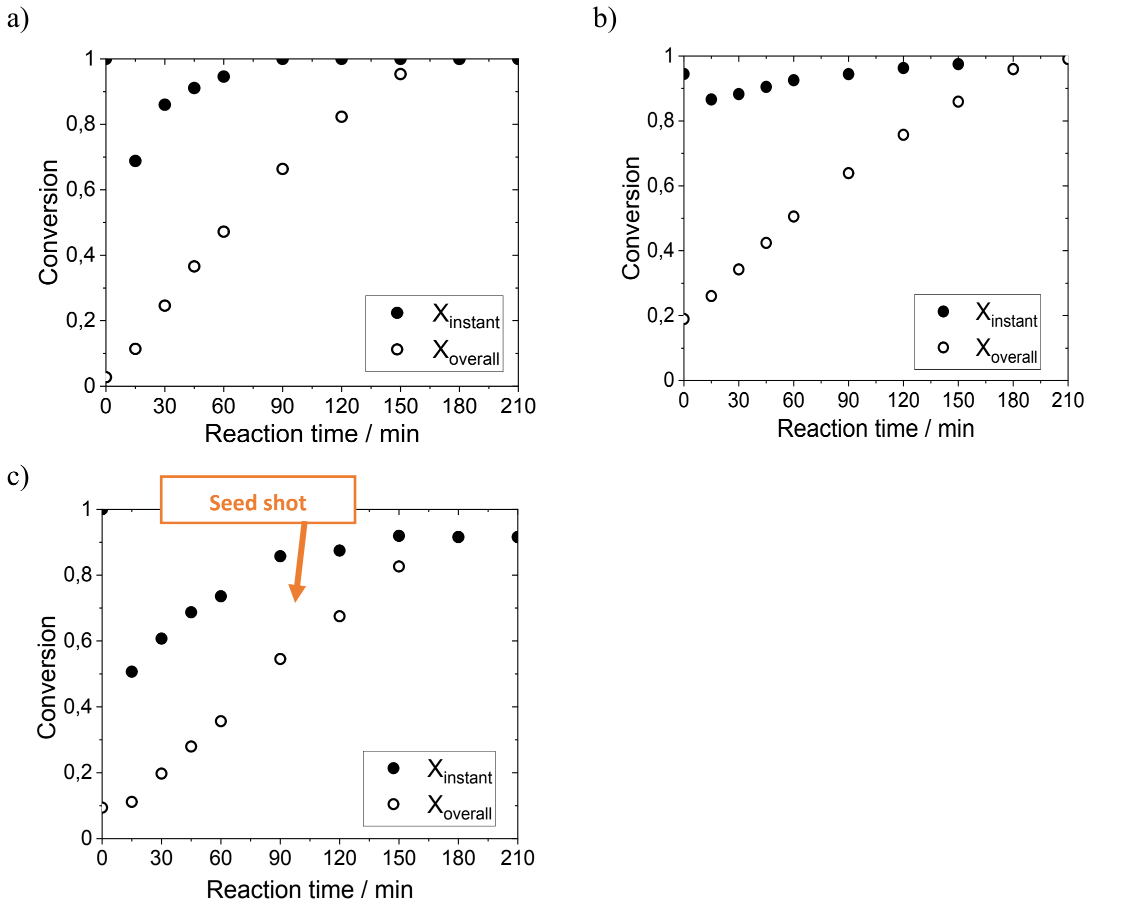

Figure SI 1. Evolution of instantaneous (filled spheres) and overall (empty spheres) conversions of reactions a) RB2, b) RB3, c) RB4.

Effect of dilution ratio in particle size measurements by OF2i

The figure below shows the retrieved average particle size for different standard latexes of PS at different dilution ratios.

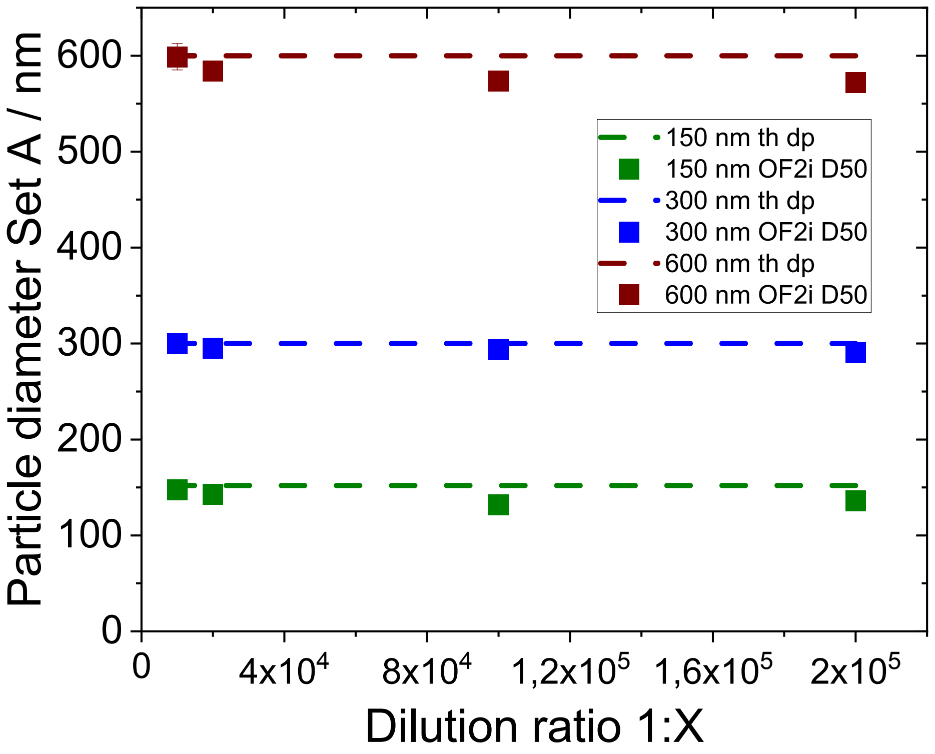

Figure SI 2. Effect of dilution ratio in particle size measured by OF2i.
